# BTK Is the Target That Keeps on Giving: A Review of BTK-Degrader Drug Development, Clinical Data, and Future Directions in CLL

**DOI:** 10.3390/cancers17030557

**Published:** 2025-02-06

**Authors:** Ross T. Salvaris, Jamie Brennan, Katharine L. Lewis

**Affiliations:** 1 Department of Hematology, Monash Health, Clayton, VIC 3168, Australia; ross.salvaris@monashhealth.org; 2 School of Clinical Sciences at Monash Health, Monash University, Clayton, VIC 3800, Australia; 3 Department of Hematology, Sir Charles Gairdner Hospital, Nedlands, WA 6009, Australia; jamiegraybrennan@gmail.com; 4 School of Medicine, University of Western Australia, Crawley, WA 6009, Australia

**Keywords:** CLL, BTK, BTK degraders

## Abstract

Effective available treatment options for patients with chronic lymphocytic leukemia/small lymphocytic lymphoma (CLL/SLL) who relapse after becoming refractory to both a covalent Bruton Tyrosine Kinase inhibitor (cBTKi) and a B cell leukemia/lymphoma 2 inhibitor (BCL2i) remain limited, and prognosis is very poor. Emerging areas of drug development include cellular therapies such as chimeric antigen receptor T-cell therapy and bispecific antibodies. However, cost, accessibility, toxicity, and the need for either prolonged or repeated hospitalization prevent universal application of these therapies. Given this area of unmet clinical need, we present this review article on Bruton Tyrosine Kinase (BTK) degraders in patients with CLL/SLL. We focus on their development as a drug class, the up-to-date clinical data available, as well as future directions. BTK protein degraders are a novel drug class with an alternate mechanism of action (MOA), compared to cBTKis and non-covalent BTKis (ncBTKis), causing ubiquitination of BTK, thereby leading to its degradation through the proteasome. Encouraging pre-clinical data show that this MOA allows BTK protein degraders to overcome common BTK mutations. We focus on four agents which are under investigation in B-cell malignancies in early clinical trials: BGB-16673, NX-2127, NX-5948, and AC676. Preliminary data suggest a comparable safety and toxicity profile between agents across this drug class with many patients on phase 1 trials deriving durable clinical benefit. Optimal sequencing of BTK degraders in the therapeutic landscape of CLL/SLL treatment is yet to be established. Further trials investigating these agents in combination with other targeted CLL agents may help to further understand their applicability. An effective, tolerable oral class of drugs would be invaluable in the treatment of patients with multiply relapsed CLL/SLL.

## Simple Summary

Oral therapies targeting receptors in a blood cancer called chronic lymphocytic leukemia (CLL) have transformed outcomes with for those with this disease. However, for patients who experience progression of their cancer after these therapies, there are very limited effective treatment options available. Bruton’s tyrosine kinase (BTK) degraders are a new class of medications that have potential to be effective in this scenario. This review describes the mechanism of action of BTK degraders, and their pre-clinical and clinical development to date, including emerging clinical trial data. Potential future directions for the development of these agents is also discussed.

## 1. Introduction

Chronic lymphocytic leukemia (CLL) is a disease where an understanding of the underlying pathobiology has led to the development of effective novel drug targets. Impaired apoptosis and aberrant B cell receptor signaling (BCR) are key abnormalities in CLL cells. With the advent of Bruton Tyrosine Kinase inhibitors (BTKi), the BCR pathway, which promotes cell growth, differentiation, and survival, can be successfully targeted, with resultant inhibition. Overexpression of the antiapoptotic protein B cell leukemia/lymphoma 2 (BCL2) leads to an accumulation of malignant cells due to an inability to undergo apoptosis. BCL2 inhibitors (BCL2is) reverse this abnormality, allowing CLL cells to undergo cell death.

With the advent of these new agents, chemotherapy is now less commonly selected for treating patients with CLL. However, patients who relapse after treatment with both a BTKi and a BCL2i (‘double refractory’) still have limited effective treatment options available. The poor prognosis in these patients highlights the need for effective novel therapies. Although early efficacy data are encouraging for cellular therapies, the cost of these therapies, their toxicity profile, and the requirement for either extended hospitalization or frequent administration may hamper their utility in routine clinical practice. Additionally, patients with CLL are frequently older than 70 years of age and often have co-morbidities that may limit their ability to tolerate these therapies. Novel, orally administered therapies with a favorable toxicity profile are needed to better treat this group of patients. Novel BTK protein degraders are an exciting drug class that may fulfill this unmet need in CLL.

This review highlights this area of unmet need in CLL, the mechanism of action of BTK protein degraders, a thorough description of the drug development, and the pre-clinic data supporting the early clinical trials in patients with B cell malignancies. The toxicity and efficacy data from these trials will be discussed, as well as potential future directions of BTK protein degraders. Whether these agents will be used alone or in combination, and how they will be sequenced with other therapies treatment is yet to be established.

## 2. Area of Unmet Need in Patients with Double Refractory CLL

With the advent of BTKis and the BCL2i venetoclax, the treatment landscape for patients with CLL has shifted markedly in both the frontline and relapsed setting. In countries where these agents are available, immunochemotherapy (ICT) is no longer used to treat patients with CLL due to superior outcomes in patients treated with these agents [1,2,3]. However, these remain costly and, consequently, access varies in different countries and regions [4]. In the modern treatment era, if available, the first two lines of therapy, namely initial treatment and at first relapse, will preferentially now include a covalent BTKi (cBTKi) and a BCL2i, either sequentially or in combination. BTKis are given as continuous therapy in both the frontline and relapse settings unless combined with venetoclax. In the frontline setting, time-limited venetoclax may be combined either with Obinutuzumab (VenO) or with a BTKi such as ibrutinib [5,6,7]. When venetoclax is used in the relapsed setting, it is routinely combined with rituximab in a fixed-duration regimen [8].

Whether patients who receive time-limited therapy as frontline therapy can be successfully re-treated at first relapse with the same regimen is yet to be fully established. Promising early data from the CAPTIVATE and IMPROVE studies suggest that retreatment after progression, which was only required more than two years after completion of primary therapy in the majority of patients, with either ibrutinib, venetoclax, or both agents, may lead to an overall response rate (ORR) of ~80–85% [9,10]. This is also being explored in the phase 2 ReVenG study (NCT04895436) which is assessing retreatment with VenO at first relapse.

‘Double refractory’ is a term coined to describe patients with CLL who have progressed and are no longer responsive to both cBTKi and venetoclax-based treatment. To separate patients with CLL who are double class exposed, where retreatment strategies may be appropriate, from those who are double refractory, Aronson et al. proposed a definition of double refractory disease where patients progressed whilst on a BTKi and whilst on or within 24 months of venetoclax [11]. The prognosis in these patients is very poor. Furthermore, they have high rates of Richter’s transformation (RT). In a retrospective series of 17 patients with a median of four prior lines of therapy who were resistant to both venetoclax and a cBTKi, the median overall survival (OS) was very short at 3.6 months [12]. Of these patients, thirteen patients died from disease progression with eight developing RT.

Disease progression during BTKi therapy is usually due to the development of BTK resistance. The most frequently observed mechanism of resistance with continuous cBTKi exposure in CLL, is the BTK Cys481Ser (C481S) mutation which renders cBTKi ineffective. To overcome this, non-covalent BTKi (ncBTKi), such as pirtobrutinib and nemtabrutinib, have been developed, which exert their effect by binding to non-C481 sites of BTK. Efficacy data have recently been presented from the phase 1/2 BRUIN study, where pirtobrutinib was administered to 282 patients with CLL/SLL who were previously exposed to a cBTKi and had either become refractory or developed intolerance. In 128 patients who had previously received both a cBTKi and a BCL2i, the ORR including partial response (PR) with lymphocytosis was 79.7% with a median duration of response (mDoR) of 14.8 months and median progression free survival(mPFS) of 15.9 months [13]. Nemtabrutinib also demonstrates efficacy in patients previously exposed to both a cBTKi and BCL2i. In the phase 1/2 BELLWAVE-001 trial, 57 patients were treated at the recommended phase 2 dose of 65 mg. There were 24 patients (42%) who had both prior cBTKi and BCL2i treatment with an ORR of 58%, mDOR of 8.5 months, and mPFS of 10.1 months [14].

Chimeric antigen receptor (CAR) T-cell therapy, and T-cell engaging antibodies, have also resulted in efficacy in early phase clinical trials where these agents are being investigated in patients with double refractory CLL. The TRANSCEND-CLL-004 study evaluated lisocabtagene maraleucel (liso-cel), a CD19-directed autologous CAR T-cell product, in patients with CLL who had received at least two prior lines of therapy including a BTKi [15]. Of 137 enrolled patients, 117 patients received liso-cel. Of these patients, 70 patients had received both prior BTKi and venetoclax. The ORR was 44% and the rate of complete response (including with incomplete marrow recovery) was 20%, with a mPFS of 11.9 months and mDOR of 35.3 months. Notably, higher rates of cytokine release syndrome (85%) and neurotoxicity (45%) were reported compared with patients with large B cell lymphoma receiving the same product in other trials and routine clinical practice [16,17]. Utility of CAR T-cell therapy in this patient population may also be hampered by rapid disease kinetics and limited effective options for use as bridging therapy during the product manufacturing period. Nonetheless, based on data from the TRANSCEND-CLL-004 study, liso-cel was approved by the FDA in March 2024 [18].

Epcoritamab is a subcutaneously administered CD3 × CD20 bispecific T-cell antibody that has been investigated in patients with CLL who have received at least two prior lines of therapy including a cBTKi. The most recent update reports outcomes for 40 patients (n = 23 expansion cohort, n = 17 optimization cohort). The optimization cohort evaluated the effect of adjustments to supportive care, including enhanced CRS prophylactic measures, on the safety of epcoritamab delivery. The ORR in the 23 expansion patients was 61% (CR 39%), with a mPFS of 12.8 months [19]. Cytokine release syndrome (CRS) was frequent (96%, the majority grade 1–2, with 17% grade 3 in the expansion cohort, and 0% grade 3 in the optimization cohort). Further data are required to evaluate the tolerability of this treatment in the CLL population, and the durability of treatment response.

## 3. The Role of the B-Cell Receptor and Targeting BTK in CLL

BTK is a cytoplasmic tyrosine kinase that is overexpressed in B-cell malignancies. BTK has an important role in B-cell activation, proliferation, signaling, and survival. BTK functions downstream of the BCR which is a cell-surface immunoglobulin and is activated upon antigen binding, inducing phosphorylation. BTK is recruited to the cell membrane with resultant autophosphorylation, leading to full activation and allowing BTK to phosphorylate its downstream target of phospholipase- Cγ2 (PLCG2). This activates pro-survival mechanisms and growth through nuclear factor kappa-light-chain-enhancer of activated B cells (NF-κB) transcriptional activity and the RAS-RAF-MEK-ERK pathway.

As discussed, cBTKis, ibrutinib, acalabrutinib, and zanubrutinib, have reshaped the treatment landscape for patients with CLL. These small molecules bind irreversibly to the cysteine 481 residue of BTK. The most frequent cause of resistance to these cBTKi in CLL is an acquired BTK mutation and substitution at this C481 site impeding BTKi binding. To overcome this mechanism of resistance, a novel class of ncBTKi, including pirtobrutinib and nemtabrutinib, have been designed.

Despite the promising efficacy of ncBTKis, a range of BTK mutations occur in patients with CLL who can develop subsequent progression on these agents, including gatekeeper mutations (T474I/F/S/L/Y), C381S/R/Y, kinase-impaired (L528W), and other mutations proximal to the ATP-binding pocket (e.g., D539A, V416L, Y545N, and A428D) [20]. The BTK L528W mutation is enriched in patients treated with zanubrutinib and can confer cross-resistance to pirtobrutinib [21]. Additionally, non-BTK mutations may lead to alternative mechanisms of resistance to ncBTKis including mutations in *TP53*, *PLCG2*, *PIK3CA*, and *BCL2*. Table 1 summarizes the common mutations seen in patients with CLL exposed to cBTKi and ncBTKis and the sensitivity of BTK degraders.

## 4. PROTACs and Early Drug Development

Over two decades ago, Crews and collaborators introduced the concept of proteolysis targeting chimera (PROTAC) molecules [27]. The first fully synthetic PROTAC, called Protac-1, was initially patented in 1999 and later published in 2001. The discovery of a peptide from the hypoxia inducible factor 1-α (HIF-1 α) subunit that bound the E3 ligase von Hippel–Lindau (VHL) tumor suppressor gene led to the design of cell penetrating PROTACs [27,28]. This was then used to target and induce degradation of androgen receptors (AR) in cancer cells. The potential for these molecules to have therapeutic potential was appreciated in 2019, with the entry of two first-in-human (FIH) trials: the PROTACs ARV-110 and ARV-471, targeting the AR in heavily pretreated patients with metastatic, castration resistance prostate cancer, and estrogen receptor (ER) in patients with ER-positive breast cancer, respectively [29,30]. Encouraging efficacy was observed across phase 1 and 2 studies with these agents.

This development occurred alongside the discovery of the E3 ligase cereblon (CRBN) which was identified as the target for thalidomide and its analogues [31]. These agents are considered early PROTACs and since 2015, VHL and CRBN E3 ligases have been widely used to develop small molecule PROTACs [32]. These immunomodulatory (IMiD) agents are now widely used therapeutically for myeloma and myelodysplastic syndrome. It was established that PROTACs based on VHL-1 and IMiDs were exceptionally potent and led to rapid degradation of substrates [32]. These collective findings heralded the development of PROTACs as therapeutics.

**Figure 1:**
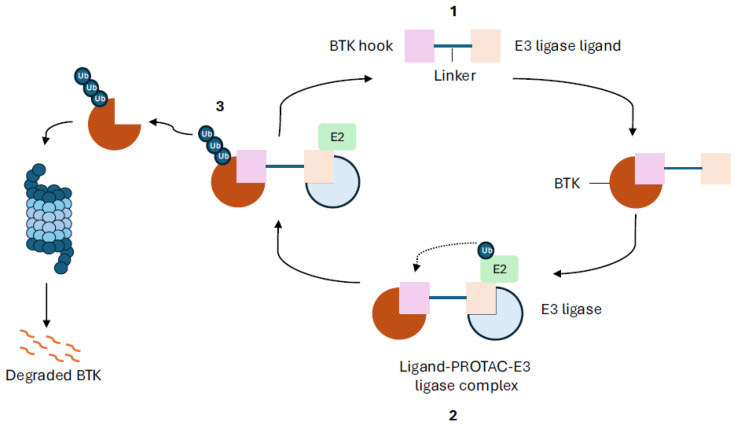
**BTK degrader mechanism of action.** Bruton tyrosine kinase (BTK) degraders cause degradation of the BTK protein through the ubiquitin–proteasome system. **1**, Degraders consist of three components: a ligand that binds to BTK (“BTK hook”) and a ligand that binds an E3 ligase (e.g., cereblon). **2**, When the degrader binds to BTK, thereby forming a tertiary complex, it leads to ubiquitination. **3**, Ubiquitination of BTK leads to its degradation by the proteasome.

The development of PROTACs to target BTK degradation in B-cell malignancies has evolved since 2018 due to their potential to overcome acquired BTK inhibitor resistance mutations which frequently occur in B-cell malignancies. Their unique pharmacological properties, including enhanced target selectivity, rapid and sustained depletion of targets, and greater potency, may lead to several potential benefits of PROTAC BTK-degradation over inhibition [30]. Additionally, unlike BTKis, PROTACs can degrade the entire BTK protein, and as such influence undruggable protein targets such as transcription factors, non-enzymatic proteins, and scaffolding, and their bifunctional structure can also be optimized to increase target selectivity [33]. Figure 1 demonstrates the mechanism of action of PROTACs (BTK degraders).

Traditional enzyme inhibitors rely on ‘occupancy driven’ pharmacology to exert their therapeutic effect, and as such always require the presence of the ligand to inhibit the target protein. In contrast, protein degraders work via ‘event driven’ pharmacology, meaning a transient binding event is enough to trigger protein degradation. This means that even low-affinity protein of interest (POI) ligands can be included in the PROTAC complex, with resultant efficient target degradation [32].

## 5. Pre-Clinical Data of BTK-Targeting PROTACs

Pre-clinical data for the first specifically designed BTK-targeted PROTACs were reported by Huang et al. [34]. These compounds, CJH-005-067 and DD-04-015, incorporated biologic agents based on the ncBTKis bosutinib (a BCR-ABL kinase inhibitor also known to inhibit BTK) and RN486 (a highly selective BTK inhibitor), respectively, along with a common pomalidomide-derived E3 ligase-binding component [35,36]. Both compounds significantly reduced BTK expression within 4 h in acute monocytic leukemia cell lines. DD-04-015 was particularly effective at around 100 nM, though it exhibited a marked “hook effect”—a reduction in BTK down-modulation at higher concentrations (10 μM) [37].

With respect to the C481S acquired resistance mutation observed during BTK inhibition, the first analysis of PROTACs targeting this were described by Buhimschi et al. in 2018 [38]. These compounds were MT-540 and MT-541, which reduced BTK expression in cells derived from Burkitt’s lymphoma. This study also demonstrated that PROTACs designed to target BTK using a VHL-binding ligand showed limited effectiveness in degradation. This indicates that successful targeting of BTK relies on the choice of a suitable E3 ligase binding component. Sun et al. described a small collection of BTK-targeted PROTACs, structurally related to ibrutinib that did not possess the acrylamide group, as well as an alternative BTK inhibitor, sperbrutinib [39,40]. These PROTACs incorporated either pomalidomide (targeting CRBN) or RG-71120-derived (targeting mouse double minute homologue 2) E3-binding elements. Overall, PROTACs containing pomalidomide showed greater potential.

Early data from FIH trials of BTK protein degraders demonstrate the unique pharmacological properties of PROTACs [41,42,43,44]. For example, it was proposed that a single PROTAC molecule can lead to repeated degradation, meaning the elimination of multiple proteins per molecule. When the POI and the E3 ligase bind, the PROTAC induces ubiquitylation of the POI and its degradation. Afterwards, the PROTAC is recycled and targets another POI, meaning PROTACs are catalytically involved in multiple rounds of targeted protein degradation. By acting like this, they remove all activity of the POI, including protein scaffolding which in turn leads to rapid, sustained, and robust inhibition of downstream signaling cascades by way of suppressing compensatory feedback activation.

## 6. BTK Degraders in Clinical Trials—Pharmacokinetics, Pharmacodynamics, and Clinical Outcomes

Novel therapies that can overcome these BTK inhibitor-induced resistance mutations may offer efficacy in patients with CLL whose disease has progressed after cBTKi and/or ncBTKi treatment. Several BTK protein degraders are currently being investigated in clinical trials including BGB-16673, AC-676, NX-5948, and NX-2127 [41,42,43,44,45]. These BTK degraders are in ongoing clinical development across a range of B-cell malignancies, with phase 1/2 studies ongoing. Preliminary safety and efficacy data have been reported for three of these BTK degraders: BGB-16673, NX-5948, and NX-2127. Currently available clinical data are discussed below and summarized in Table 2.

## 7. BGB-16673

BGB-16673 is an orally administered small molecule with an E3 ubiquitin ligase-binding moiety linked to a BTK-binding moiety [49]. Degradation of the bound BTK occurs as the E3 ligase-binding moiety binds cereblon which catalyzes ubiquitination leading to proteasome-mediated degradation. The resulting degradation of BTK prevents it activating the BCR and its downstream signaling pathway, thereby inhibiting malignant B-cell activation and growth. BGB-16673 is able to degrade both wild-type (WT) BTK as well as BTK with known mutations causing resistance to covalent and non-covalent BTKis, and in pre-clinical models demonstrated tumor suppression [49].

Pharmacokinetic studies demonstrate an elimination half-life ranging from 7.2 to 10 h in rats, and it noted the compound to be highly protein bound. It is metabolized by Cytochrome P450 3A (CYP3A), meaning that CYP3A inhibitors and inducers may be anticipated to affect BGB-16673 exposure. Other important drug–drug interactions to consider are those of pH lowering medications, given BGB-16673 has pH-dependent solubility. Early pre-clinical data also support that BGB-16673 exhibits a longer duration of response when compared with ibrutinib and pirtobrutinib, suggesting a disconnect between the pharmacodynamics and pharmacokinetics of this molecule, suggesting it may provide a lasting therapeutic effect after it has been eliminated from the circulation [50].

BGB-16673 is being investigated in an ongoing phase 1/2 dose escalation and expansion study (CaDAnCe-101; NCT05006716). Eligible patients have selected B-cell malignancies: marginal zone lymphoma (MZL), follicular lymphoma (FL), mantle cell lymphoma (MCL), CLL/SLL, Waldenstrom’s macroglobulinemia (WM), diffuse large B-cell lymphoma (DLBCL), and RT; and have relapsed following, or were refractory to, at least two prior lines of therapy, which must include a cBTKi for disease types where this is approved. In the dose-escalation portion of the study, patients were planned to receive BGB-16673 at doses between 50 and 600 mg once daily. Treatment was administered in 28-day cycles until disease progression, unacceptable toxicity, or withdrawal.

Pharmacodynamic data revealed substantial reduction in BTK protein levels in peripheral blood and tumor tissue including at the lowest dose (50 mg) [51].

Data for patients with CLL/SLL in the CaDAnCe-101 study were recently presented [44]. Forty-nine patients with relapsed/refractory CLL or SLL were enrolled and received doses between 50 and 500 mg once daily of BGB-16673. At data cutoff, forty (82%) remain on treatment, with nine having discontinued treatment for progressive disease (n = 4), adverse event (n = 4), and investigator discretion (n = 1, for persistent low-grade arthralgia). Patients were heavily pretreated, with a median number of prior therapies of 4 (range 2–10). Median follow-up was 4.6 months (range 0.3–19.8). Many patients had high-risk CLL characteristics (unmutated IGHV, 82%; TP53 mutation or del(17p), 60%; or complex karyotype, 47%), and 15/47 (32%) had a documented BTK mutation prior to enrolment. Ninety-two percent (n = 45) had received a cBTKi, 76% (n = 37) had received a cBTKi and BCL2i, and 22% (n = 11) had received a cBTKi, BCL2i, and ncBTKi. The ORR was 72% in response evaluable patients (31/43) including complete responses (n = 2, 200 mg cohort). Responses were similar across high-risk patient groups (see Table 1), with responses also observed in patients with BTK and PLCG2 mutations.

Adverse events (AE) are summarized in Table 1 and align with the adverse event profile of cBTKi and ncBTKi. Most frequent AEs were fatigue (33%), contusion (29%), anemia (22%), diarrhea (22%), and neutropenia (22%). Most frequent grade ≥ 3 AEs were neutropenia (20%) and pneumonia (12%). No hypertension or atrial fibrillation/flutter was reported to date. One dose limiting toxicity was observed at a dose of 200 mg, a grade 3 rash which did not recur upon treatment rechallenge at the same dose, and treatment has been able to continue. Safety and efficacy from the CaDAnCe-101 study for patients with other B-cell malignancies has also been presented, with a comparable adverse event profile [52].

## 8. NX-2127

NX-2127 is BTK protein degrader that recruits cereblon to degrade BTK. Additionally, NX-2127 has activity akin to other IMiDs via the ubiquitination of targets including Ikaros and Aiolos resulting in increased T-cell activation [53]. NX-2127 degrades both wild type (WT) and C481-mutated BTK protein. This dual activity of IMiD-like activity and BTK degradation may offer efficacy in patients with CLL with acquired BTK mutations [54].

Data from the NX-2127 trial demonstrated this where it exhibited strong and persistent BTK degradation in all patients treated with this molecule, irrespective of their initial BTK levels or the dosage of NX-2127 administered [43].

NX-2127 differs from the other BTK degraders, inducing BTK degradation via recruitment of cereblon, with subsequent dual BTK degrader and immunomodulatory-like activity. In the ongoing FIH NX-2127-001 study (NCT04830137), patients with relapsed/refractory B-cell malignancies received oral NX-2127 at doses of 100–300 mg once daily until progressive disease, unacceptable toxicity, or withdrawal. Early clinical data were recently presented [41]. Forty-seven patients were enrolled, including twenty-nine with CLL/SLL. Other histologies included DLBCL (n = 5), MCL (n = 5), MZL (n = 3), WM (n = 3) and FL (n = 2). Again, patients were heavily pretreated with median prior lines of therapy 4 (range 2–10) for non-Hodgkin lymphoma subtypes and five (range 2–11) for CLL. All patients with CLL had received a prior BTKi (either covalent, non-covalent, or both) and 76% had received a BCL2i. BTK and BCL2 mutations were reported at enrolment, although the proportion of patients with mutations is not described. There were two dose limiting toxicities: neurological/cognitive disturbance of uncertain etiology (n = 1) and neutropenia (n = 1). Granular details regarding these events have not yet been reported. Most frequently reported adverse events were fatigue (48.9%), neutropenia (42.6%), hypertension (36.2%), and contusion (27.7%) with the most frequent grade ≥ 3 events being neutropenia (38.3%), hypertension (14.9%), and anemia (12.8%). Grade 3 atrial fibrillation was observed in 6.4%, with a further 6.4% experiencing grade ≤ 2 atrial arrythmias. The median follow-up in this study was 9.5 months (range 0.1–24.3). Rapid, substantial, and sustained BTK degradation was again noted in all patients, even at the lowest dose level. ORR for evaluable patients with CLL was 9/24 (37.5%), all partial responses, with a further 11/24 (45.6%) exhibiting stable disease. At the time of reporting, dose expansion has not been initiated in CLL, but expansion cohorts were ongoing in DLBCL and MCL.

## 9. NX-5948

NX-5948 is an oral small molecule that causes BTK degradation via the cereblon E3-ubiquitin ligase complex but has been designed to avoid Ikaros and Aiolos degradation, therefore not possessing IMiD activity [45]. In addition to its degradation of WT and mutant forms of BTK, NX-5948 can cross the blood–brain barrier and degrade BTK intracranially. NX-5948 has demonstrated pre-clinical efficacy in mouse models with an intracranial BTK-dependent activated-B-cell-diffuse large-B-cell lymphoma cell line, TMD8 [45]. NX-5948 led to decreased intracranial tumor burden as well as improved survival.

NX-5948-301 (NCT05131022) is a phase 1 dose escalation study of NX-5948 in patients with relapsed/refractory B-cell malignancies who have received at least two prior lines of therapy. Since NX-5948 was able to cross the blood–brain barrier in pre-clinical studies, patients with active CNS involvement of their disease were eligible. Data from the phase 1 study have been recently presented [42]. Forty-six patients had received treatment with NX-5948, at doses ranging from 50 to 600 mg. Treatment was administered orally, once daily in 28-day cycles until progressive disease, unacceptable toxicity, or withdrawal. This cohort of 46 patients included 16 with CLL. The median prior number of prior therapies was four (range 2–14), with 14/16 CLL patients having received a BCL2i and either a cBTKi or ncBTKi. BTK and TP53 mutations were present in some patients at study enrolment (5/12 for each, respectively). Median follow-up was 3.4 months (range 0.2–20.2). Pharmacodynamic data revealed rapid, sustained BTK degradation in all patients. The ORR in response evaluable CLL patients was 70% (7/10) with all responses being a partial response.

The adverse event profile was again, as anticipated, a BTK pathway targeting agent, with the most frequent AEs being contusion (39%), thrombocytopenia (37%), and neutropenia (26.1%). There was one dose limiting toxicity, a rash occurring at 450 mg, which did not occur upon rechallenge. There have been no episodes of atrial fibrillation/flutter to date.

## 10. AC676

AC676 is a chimeric degrader that targets and degrades BTK using the protein–protein-interaction-targeting chimeras platform. No clinical data have yet been reported for this agent. However, a phase 1 dose escalation trial is ongoing for patients with relapsed/refractory B-cell malignancies including CLL, SLL, MCL, WM, FL, non-GCB DLBCL, and MZL (NCT05780034). AC676 is administered orally, once daily on a 28-day cycle, with planned evaluable dose levels ranging from 50 to 600 mg.

## 11. Summary of Clinical Data

Early clinical data in this drug class are encouraging, with efficacy signals in phase 1 studies across a range of B-cell malignancies, including CLL. Although follow-up for all three agents remains short at this time, many patients remained on therapy at the time of reporting, with ongoing clinical benefit. At this early stage of development, the safety and toxicity profile appear comparable between agents across this drug class. The well documented adverse events observed with BTK pathway inhibition predominate, including neutropenia, infection, contusion, and fatigue. There are early signals of efficacy with documented objective disease responses for all agents, across a range of histologies.

A longer follow-up is required to evaluate the durability of these responses. Additionally, during early BTKi development, some adverse events (e.g., hypertension and atrial fibrillation) progressively emerged with more prolonged duration of therapy. Current follow-up in these early studies investigating BTK degraders remains too short to appreciate long-term adverse event evolution.

Despite limited follow-up at this time, the early efficacy data for BTK degraders in relapsed/refractory CLL are compelling and have led to the granting of FDA fast track designation status for both NX-5948 and BGB-16673 in this indication [55,56]. Phase 1 and 2 studies are ongoing, and studies investigating these agents in combination with other therapies are also in development (NCT06634589).

## 12. Future Directions for BTK Degraders

The early clinical data above suggest that BTK degraders may be an effective drug class for the treatment of CLL, especially valuable for the patients with ‘double refractory’ CLL/SLL who have limited available therapeutic options. However, although many patients treated in the early clinical trials with these agents remain on therapy with ongoing CLL control, some patients treated with these compounds have unfortunately already experienced disease progression. Current understanding of the mechanisms for resistance with BTK degraders remains immature, but there is emerging data to suggest that despite their activity degrading wild-type and mutant-BTK, BTK degrader activity may still be vulnerable to BTK mutations. A recent case report describes the clinical course and mutational evolution in a patient with CLL treated with BGB-16673 [26]. Serial mutation testing through sequential lines of therapy documented the acquisition of a new A428D BTK mutation during treatment with BGB-16673, with subsequent clinical disease progression. It is noteworthy that another degrader in clinical development (NX-2127) exerts its action by interacting with the A428 site on BTK, and a similar mutation and pattern of disease resistance may be anticipated with this agent, although to our knowledge this has not yet been reported. Current and future clinical studies of BTK degraders may be designed to facilitate investigation of the mechanisms of emergent resistance.

With emerging preliminary data on resistance and with multiple pathways by which B-cell malignancies may become resistant to targeted therapies, it is hypothesized that targeting multiple B-cell pathways simultaneously, or targeting BTK simultaneously via multiple mechanisms, may offer synergistic antitumor activity compared with degrader monotherapy. Combining other therapeutic agents with degraders may result in complementary actions to increase response rates and deepen or prolong responses to treatment. A phase 1b/2 trial utilizing BGB-16673 in combination with other agents is underway (NCT06634589), with sub-studies designed to evaluate different therapeutic combinations across a range of B-cell malignancies. The first planned sub-studies will include BGB-16673 plus sonrotoclax, and BGB-16673 plus zanubrutinib across a range of B-cell malignancies, including but not limited to WM, CLL, MCL, and MZL. The trial design allows for the incorporation of additional therapeutic combination sub-studies in the future, as data emerge for various B-cell targeting agents. There are currently no active studies investigating NX-2127 or NX-5948 in combination regimens, however, with the biological rationale for synergism between B-cell targeting agents, these compounds may also have potential to be developed in similar combinations in future.

## 13. Conclusions

In patients with CLL/SLL who relapse after treatment with cBTKis and ncBTKis, the development of BTK degraders shows that BTK may remain a valid drug target. BTK degraders’ unique mechanism of action allows efficacy despite the acquisition of common BTK mutations as shown in pre-clinical data and suggested by the encouraging efficacy observed to date in the early-phase clinical trials. Early safety data suggest that these agents are tolerable, but longer-term follow-up is required to assess for treatment emergent adverse events that may only emerge with prolonged exposure, as previously observed with BTK inhibitors. Whether these agents will be most effectively employed as monotherapy or in combination with other novel agents, such as BCL2 inhibitors or cBTKis, remains to be established. Upcoming clinical trials will further explore combination therapy. Optimal sequencing in relation to other agents and cellular therapies, if available, will be an area of substantial debate. This exciting class of drugs may offer patients with double refractory CLL/SLL an effective, tolerable therapy in an area of unmet need.

## Figures and Tables

**Table 1:** BTK mutations after covalent and noncovalent BTK inhibitor exposure and relative BTK degrader sensitivity.

BTK Mutations Leading to Covalent BTKi Resistance	BTK Mutation Enriched by Exposure to Specific cBTKi	Pirtobrutinib Sensitive?	BTK Mutations Leading to Pirtobrutinib Resistance	BTK Degrader Sensitive?
C481S [22]	Ibrutinib/zanubrutinib/acalabrutinib	Yes [13]	L528W [20,23]	Yes
L528W [24]	Zanubrutinib	No [20]	T474I [20,23]	Yes
T474I [25]	Acalabrutinib	No [20]	A428D [20,23]	?BGB-16673 resistant [26]
			M437R [20,23]	Yes
			V416L [23]	Yes
			C481F [23]	Yes
			C481R [20]	Yes

BTKi, Bruton tyrosine kinase inhibitor; cBTKi, covalent BTK inhibitor; ?, query.

**Table 2:** Summary of data for BTK degraders in clinical development.

Compound	Trial	CLL/SLL pts, n	CLL Features	Regimen	Prior Therapies (Range)	Efficacy (CLL) n (%)	Toxicity n (%)			FU Months (Range)	Notes
							Adverse Event	All	G ≥ 3		
BGB-16673 [46]	CaDAnCe-101 (NCT05006716)	49	IGHVu 82% TP53 mut or 17p—63% Complex karyotype 47% Known BTK mutation 32%	QD Oral Continuous Dose levels 50–600 mg	Median 4 (2–10) cBTKi: 92% BCL2i: 86% ncBTKi: 24% 22%	ORR 38/49 (78%) ORR at 200 mg dose = (15/16) 94% CR 2/49 (4%) (both at 200 mg) Responses in high risk and patient with BTK mutations	Fatigue Contusion Anemia Diarrhea Neutropenia No AF, No G ≥ 3 hypertension	17 (35%) 14 (29%) 11 (22%) 13 (27%) 11 (22%)	1 (2%) - 1 (2%) 1 (2%) 10 (20%)	Median 7.9 (0.3–23.1)	Other eligible B-cell histologies: MZL, FL, MCL, WM, DLBCL, RT N = 1 dose limiting toxicity; grade 3 rash—resolved to grade 1 and patient continued on treatment without recurrence. Median time to first response = 2.8 months range (2.6–8.3) 40/49 (82%) remain on treatment
NX-5948 [47]	NX-5948-301 (NCT05131022)	34	Known BTK mutation 42% TP53 mutation 48.4% PCLG2 mutation 17%	QD Oral Continuous Dose levels 50–600 mg	Median 4 (2–14) cBTKi/ncBTKi + BCL2i: 88%	ORR 30/34 (76.4%) CR 0/30 (0%)	Contusion Platelet decreased Neutropenia No AF	15 (44.1%) 8 (23.5%) 6 (17.6%)	- 1 (2.9%) 5 (14.7%)	Median 4.7 (0.7–17.0)	Pre-clinical data—can cross blood-brain-barrier → patients with CNS involvement eligible (n = 6 NHL) n = 1 Grade 5 pulmonary embolism, not treatment related 10/16 = response evaluable
NX-2127 [43]	NX-2127-001 (NCT04830137)	29	BTK and BCL2 mutations in some patients	QD Oral Continuous Dose levels 100–300 mg	Median 4 (2–10) cBTKI and/or ncBTKi 100% BCL2i 76%	ORR 9/24 (37.5) CR 0/24 (0%) SD 11/24 (46%)	Fatigue Neutropenia Hypertension Anemia Atrial arrythmia	23 (49%) 20 (43%) 17 (36%) Unk 3 (6%)	- 18 (38%) 7 (15%) 6 (13%) 3 (6%)	Median 9.5 (0.1–24.3)	iMID-like activity 24/29 = response evaluable
AC676 [48]	AC676-001 NCT05780034	NA	NA	QD Oral Continuous Dose levels planned 50–600 mg	NA	NA	NA	NA	NA	NA	NA

CLL/SLL, chronic lymphocytic leukemia/small lymphocytic lymphoma; n, number of patients; G, grade; FU, follow-up; BTK, Bruton’s tyrosine kinase; QD, once daily; ORR, overall response rate; CR, complete response rate; AF, atrial fibrillation or flutter; cBTKi, covalent BTK inhibitor; ncBTKi, non-covalent BTK inhibitor; BCL2i, B-cell lymphoma 2 inhibitor; MZL, marginal zone lymphoma; FL, follicular lymphoma; MCL, mantle cell lymphoma; WM, Waldenstrom’s macroglobulinaemia; DLBCL < diffuse large B-cell lymphoma; RT, Richter’s transformation; CNS, central nervous system; NHL, non-Hodgkin lymphoma; SD, stable disease; iMID, immune modulatory; IGHVu, IGHV unmutated; and TP53 mut, TP53 mutation present.
